# Drain management after pancreatoduodenectomy: risk-stratified dynamic algorithm

**DOI:** 10.1093/bjsopen/zrag068

**Published:** 2026-06-26

**Authors:** Giampaolo Perri, Elisa Bannone, Isabella Frigerio, Riccardo Pellegrini, Martina Guerra, Roberta Vella, Alice Cattelani, Elvira Adinolfi, Umberto Cillo, Giovanni Butturini, Giovanni Marchegiani

**Affiliations:** Hepato-pancreato-biliary and Liver Transplant Surgery Unit, Department of Surgical, Oncological and Gastroenterological Sciences (DiSCOG), University of Padua, Padua, Italy; Hepato-pancreato-biliary Surgery Department, Pederzoli Hospital, Peschiera Del Garda, Italy; Hepato-pancreato-biliary Surgery Department, Pederzoli Hospital, Peschiera Del Garda, Italy; Collegium Medicum, SAN University, Lodz, Poland; Hepato-pancreato-biliary and Liver Transplant Surgery Unit, Department of Surgical, Oncological and Gastroenterological Sciences (DiSCOG), University of Padua, Padua, Italy; Hepato-pancreato-biliary Surgery Department, Pederzoli Hospital, Peschiera Del Garda, Italy; Hepato-pancreato-biliary Surgery Department, Pederzoli Hospital, Peschiera Del Garda, Italy; Hepato-pancreato-biliary Surgery Department, Pederzoli Hospital, Peschiera Del Garda, Italy; Hepato-pancreato-biliary Surgery Department, Pederzoli Hospital, Peschiera Del Garda, Italy; Hepato-pancreato-biliary and Liver Transplant Surgery Unit, Department of Surgical, Oncological and Gastroenterological Sciences (DiSCOG), University of Padua, Padua, Italy; Hepato-pancreato-biliary Surgery Department, Pederzoli Hospital, Peschiera Del Garda, Italy; Hepato-pancreato-biliary and Liver Transplant Surgery Unit, Department of Surgical, Oncological and Gastroenterological Sciences (DiSCOG), University of Padua, Padua, Italy

**Keywords:** pancreatectomy, postoperative, POPF, pancreatic, amylase, stent

## Abstract

**Background:**

The ability of drain fluid amylase (DFA) to predict pancreatic fistula (POPF) and safely guide early drain removal is unknown in high-risk pancreatoduodenectomy (HR-PD). The aim of this analysis was to develop a new risk-based algorithm for drain management after pancreatoduodenectomy.

**Methods:**

All consecutive patients undergoing pancreatoduodenectomy (2019–2024) at two institutions were included. During surgery, patients were stratified into HR-PD, defined according to the International Study Group on Pancreatic Surgery score (soft gland and duct ≤ 3 mm), and non-HR-PD. Postoperative predictors (DFA, serum amylase/lipase, C-reactive protein) were retrieved on postoperative days (PODs) 1 to 5 and correlated to POPF development using a risk-based approach. Results were validated in a retrospective cohort (2015–2019) of pancreatoduodenectomies performed at the same institutions.

**Results:**

Out of 825 screened patients, 788 were included. HR-PD (251, 32%) had higher POPF rates (50% *versus* 9%; *P* < 0.001) and worse outcomes. In HR-PD, DFA had a lower area under the curve for POPF for POD 1 (0.73 *versus* 0.85; *P* = 0.003) and POD 3 (0.74 *versus* 0.88; *P* = 0.002) compared with non-HR-PD, but similar for POD 5 (0.83 *versus* 0.86; *P* = 0.535), whereas serum amylase/lipase maintained a low area under the curve for POPF compared with non-HR-PD. The optimal criteria for POD 3 drain removal in non-HR-PD were POD 1 DFA < 200 UI/l, POD 3 DFA < 150 UI/l, and absence of elevated serum amylase/lipase on POD 1 (Youden’s index 0.674). In HR-PD, the criteria for POD 5 drain removal were POD 5 DFA < 150 UI/l and POD 5 C-reactive protein < 150 mg/ml (Youden’s index 0.593).

**Conclusion:**

In patients with an intrinsic higher risk of POPF, early predictors are less reliable, and drain management protocols should be tailored. In non-HR-PD, an early removal policy can be recommended based on DFA and serum amylase/lipase. In HR-PD, drain removal may be safely deferred until POD 5 according to DFA and C-reactive protein.

## Teaser

This multicentre study of 788 patients undergoing pancreatoduodenectomy (PD) evaluates the reliability of drain fluid amylase (DFA) as a predictor of postoperative pancreatic fistula among high-risk (HR) and non-HR-PD. We found that, whereas early drain removal guided by DFA and serum enzymes is safe in non-HR-PD, DFA accuracy is reduced in HR-PD until postoperative day 5, when combined DFA and C-reactive protein levels allow safe drain removal. These findings support a dynamic, risk-based drain management protocol tailored to pancreatic fistula risk.

## Introduction

Early drain removal is a fundamental step in the enhanced recovery after surgery pathway in patients undergoing pancreatoduodenectomy (PD)^1^. Its implementation in postoperative care is associated with improved outcomes for postoperative pancreatic fistula (POPF), other pancreas-specific morbidity, intra-abdominal infections, and length of hospital stay (LOS)^2–4^. The timely identification of patients at the lowest risk of developing a POPF is essential for the safe implementation of early drain removal policies^5^.

Several protocols have been proposed to guide decision-making, most of which are based on assessing drain fluid amylase (DFA) during the early postoperative days (PODs)^6–10^. These protocols typically rely on definite DFA thresholds, usually associated to a high negative predictive value (NPV). However, a protocol based solely on a single DFA threshold is not suitable for all clinical scenarios. A ‘one-size-fits-all’ approach is inherently inadequate, given the significant effect of patient- and pancreas-specific risk factors as well as the dynamic nature of POPF prediction and diagnosis during the early postoperative course^8,9,11–13^. A risk-based strategy for POPF prediction and mitigation is currently the standard of care during PD, but there are no standardized or widely applied protocols for postoperative drain management specifically for patients with significantly increased POPF risk. Moreover, variables beyond DFA may be employed not only to predict POPF severity and guide appropriate management but also to inform drain removal^14–16^.

In the case of high-risk PD (HR-PD), typically defined by a soft gland and not dilated pancreatic duct^17^, early drain removal based solely on DFA could be even detrimental. Yet, no alternative reliable predictors are currently available to guide decision-making in this critical subset of patients. Other factors, such as postoperative serum hyperamylasaemia/hyperlipasaemia^18–20^ or C-reactive protein (C-RP), may help improve patient stratification for early drain removal. Moreover, a dynamic reassessment of these parameters during the first PODs may be required to improve prognostic performance.

The aim of this study was to monitor dynamically the values of DFA and other potential predictors of POPF during the early postoperative period, correlate them with the onset of POPF, and establish a new risk-adapted algorithm protocol for postoperative drain management in patients undergoing PD.

## Methods

### Study design

The study was designed as a retrospective analysis of prospectively collected data. All patients undergoing PD from 1 January 2019 to 31 December 2024 at two high-volume hepato-pancreato-biliary surgery institutions were considered eligible. The study period was the same for both institutions. The follow-up was conducted over a 90-day recovery period. The study was performed according to the STROBE guidelines^21^ (see *Supplementary material*). Validation was performed in a retrospective cohort of patients who underwent PD at the two institutions before the study period (2015–2019).

### Procedures

PD was performed according to institutional standards. During surgery, the presumed preoperative pathology, estimated blood loss, intraoperative pancreatic texture, and main pancreatic duct (MPD) size were recorded^22^. Fistula risk was assessed using the International Study Group on Pancreatic Surgery (ISGPS) four-tier classification^17^, which incorporates MPD size and gland texture. HR-PD was defined as ISGPS ‘class D’: soft gland texture with MPD size ≤ 3 mm.

All patients underwent pancreatico-jejunostomy, either single- or double-layered with duct-to-mucosa, or, less frequently, pancreatico-gastrostomy. An externalized trans-anastomotic stent (ETS) (PankreaPlus™ polyvinyl catheter, Munich, Germany) was employed as a specific mitigation strategy for POPF prevention in HR-PD and in general in case of MPD ≤ 3 mm, with a previously described surgical technique^23^. Patients where an internalized trans-anastomotic stent was employed were excluded from the present analysis.

Two open, passive drains were placed in the proximity of the pancreatic and biliary anastomoses in all patients. Early drain removal was defined as removal on PODs 1–3. According to institutional standards, early removal policies were followed in non-HR-PD patients, based on the drain fluid aspect and POD 1 DFA^3,13,24^. In all HR-PD patients, drains were left in place until POD 5, except in patients with very low output (≤ 20 cc). From POD 5 onward, in both non-HR-PD patients without early removal criteria and in all HR-PD patients, drains were periodically re-evaluated based on their appearance, output volume, amylase levels, and clinical parameters, and were removed at the surgeon’s discretion in the absence of predefined protocols.

### Postoperative POPF indicators

**Fig. 1:**
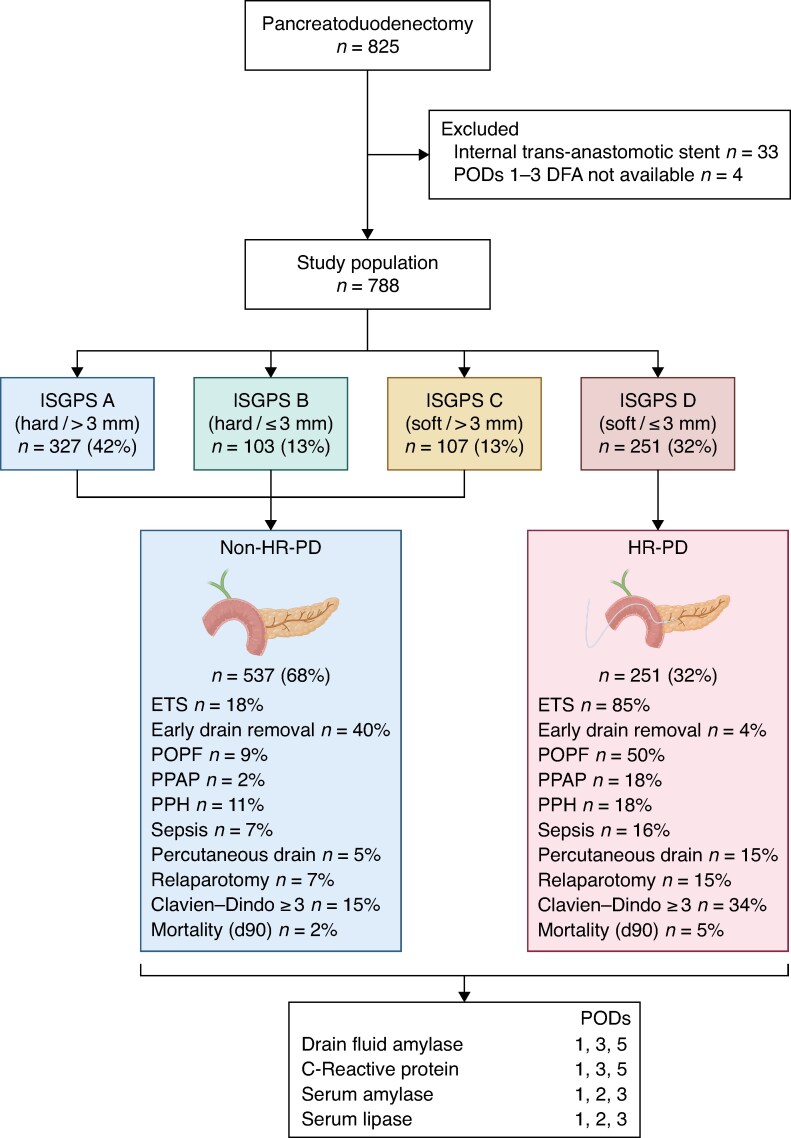
Study flowchart with intra- and postoperative outcomes POD, postoperative day; DFA, drain fluid amylase; ISGPS, International Study Group on Pancreatic Surgery; HR-PD, high-risk pancreatoduodenectomy, ETS, externalized trans-anastomotic stent; POPF, postoperative pancreatic fistula; PPAP, post-pancreatectomy acute pancreatitis; PPH, post-pancreatectomy haemorrhage.

Prospective collection of postoperative biochemical data was maintained during the entire duration of the study period and included DFA and serum C-RP (normal range 0–5 mg/ml) on PODs 1, 3, and 5, and serum pancreatic amylase (SA) (normal range 13–53 U/l) or serum lipase (SL) (normal range 5–60 U/l) on PODs 1, 2, and 3. Patients with missing DFA activity on both POD 1 and 3 were excluded from the analysis. The study flowchart is displayed in *Fig. 1*. For the retrospective validation cohort, DFA values on PODs 1, 3, and (if available) 5 were retrieved.

### Outcomes of interest

The primary outcome was the incidence of POPF, defined according to the ISGPS^25^. Secondary outcomes included the severity of POPF (grade B or C, per ISGPS^25^). All postoperative outcomes were registered prospectively, including post-pancreatectomy haemorrhage (PPH)^26^, delayed gastric emptying (DGE)^27^, postoperative hyperamylasaemia, and post-pancreatectomy acute pancreatitis (PPAP)^28^, 90-day hospital mortality and readmission, major morbidity (defined as Clavien–Dindo ≥ 3)^29^, and LOS.

### Statistical analysis

Continuous variables were expressed as median (interquartile range (i.q.r.)). Differences in variables between or among groups were tested using Student’s *t*-test for continuous variables, and the χ^2^ test or Fisher’s exact test for categorical variables. Receiver operating characteristic (ROC) curves were used to identify the optimal cut-off values of DFA with the highest performance for ruling out the occurrence of POPF, and the areas under the curve (AUC) were compared using the DeLong test for paired ROC curves. Missing-at-random variables were considered acceptable for exclusion without imputation with a threshold of ≤ 10% in the complete-case analysis. To evaluate the missing-at-random assumption, baseline characteristics of participants with complete data were compared with those of participants with missing values. Youden’s index (Youden) was used to select the most appropriate cut-off, along with the cut-off’s clinical relevance (for example, the number of patients for whom the cut-off can be applied and the POPF rate in the population selected by the cut-off). For each cut-off, sensitivity (SENS), specificity (SPEC), positive predictive value (PPV), NPV, and prevalence of patients with values above the threshold in the examined population (Prev+) were calculated. Multivariable analysis was performed using a logistic regression model, with results expressed as odds ratios (ORs) and 95% confidence intervals (c.i.). *P* values less than 0.05 were considered statistically significant. Statistical analyses were performed using STATA14 for Windows. Figures were created with BioRender.com.

## Results

A total of 825 patients were enrolled (*Fig. 1*). Of these, 33 patients who had an internalized trans-anastomotic stent were excluded. An additional four patients were excluded due to unavailable DFA measurements on both PODs 1 and 3, resulting in a final study cohort of 788 patients.

Among the included patients, 251 (32%) were considered HR-PD and 537 (68%) non-HR-PD (*Table 1* and *Table S1*). Compared with non-HR-PD, HR-PD patients had higher median body mass index (25 (i.q.r. 22–27) *versus* 24 (i.q.r. 22–27); *P* = 0.018), were less likely to have diabetes (17% *versus* 32%; *P* < 0.001), underwent jaundice palliation (46% *versus* 60%; *P* = 0.001) or neoadjuvant therapy less frequently (13% *versus* 35%; *P* < 0.001), and had a presumed diagnosis of pancreatic ductal adenocarcinoma (35% *versus* 73%; *P* < 0.001) or the need for vascular resection less frequently (9% *versus* 16%; *P* = 0.008).

**Table 1:** Intraoperative and postoperative profile of included patients, according to POPF risk

	Total (*n* = 788)	HR-PD		*P*
		No (*n* = 537, 68%)	Yes (*n* = 251, 32%)	
**Intraoperative**				
PD type				0.106
Pylorus-preserving	677 (86%)	454 (85%)	223 (89%)	
Whipple	111 (14%)	83 (15%)	28 (11%)	
Surgical approach				0.057
Open	694 (88%)	481 (90%)	213 (85%)	
Robotic	94 (12%)	56 (10%)	38 (15%)	
Pancreatic anastomosis				0.462
Pancreatico-jejunostomy	776 (98%)	530 (99%)	246 (98%)	
Pancreatico-gastrostomy	12 (2%)	7 (1%)	5 (2%)	
External trans-anastomotic stent				< 0.001
No	479 (61%)	442 (82%)	37 (15%)	
Yes	309 (39%)	95 (18%)	214 (85%)	
Vascular resection	106 (13%)	84 (16%)	22 (9%)	0.008
Operative time (min), median (i.q.r.)	405 (335–475)	400 (330–470)	420 (340–480)	0.048
Blood loss (ml), median (i.q.r.)	300 (200–400)	300 (200–400)	300 (200–450)	0.882
MPD size (mm), median (i.q.r.)	4 (3–5)	5 (4–6)	2 (2–3)	< 0.001
MPD size ≤ 3 mm	354 (45%)	103 (19%)	251 (100%)	< 0.001
Pancreatic texture				< 0.001
Hard	430 (55%)	430 (80%)	_	
Soft	358 (45%)	107 (20%)	251 (100%)	
ISGPS class				< 0.001
A	327 (42%)	327 (61%)	_	
B	103 (13%)	103 (19%)	_	
C	107 (13%)	107 (20%)	_	
D	251 (32%)	_	251 (100%)	
**Postoperative**				
POPF	173 (22%)	48 (9%)	125 (50%)	< 0.001
POPF grade				< 0.001
B	143 (18%)	36 (7%)	107 (43%)	
C	30 (4%)	12 (2%)	18 (7%)	
PPAP	57 (7%)	13 (2%)	44 (18%)	< 0.001
PPAP grade				< 0.001
POH	159 (20%)	59 (11%)	100 (40%)	
B	39 (5%)	8 (1%)	31 (13%)	
C	18 (2%)	5 (1%)	13 (5%)	
Intra-abdominal collection (deep surgical site infection)	155 (20%)	58 (11%)	97 (39%)	< 0.001
Chylous fistula	38 (5%)	32 (6%)	6 (2%)	0.029
PPH	105 (13%)	59 (11%)	46 (18%)	0.005
PPH grade				0.025
A	16 (2%)	11 (2%)	5 (2%)	
B	46 (6%)	25 (5%)	21 (8%)	
C	43 (5%)	23 (4%)	20 (8%)	
DGE	180 (23%)	99 (18%)	81 (32%)	< 0.001
DGE grade				< 0.001
A	49 (6%)	34 (6%)	15 (6%)	
B	73 (9%)	37 (7%)	36 (14%)	
C	58 (7%)	28 (5%)	30 (12%)	
Sepsis	75 (10%)	35 (7%)	40 (16%)	< 0.001
Percutaneous drainage	63 (8%)	26 (5%)	37 (15%)	< 0.001
Relaparotomy	75 (10%)	37 (7%)	38 (15%)	< 0.001
Unplanned intensive care unit admission	99 (13%)	47 (9%)	52 (21%)	< 0.001
POD of drain removal (days), median (i.q.r.)	5 (3–8)	4 (3–6)	10 (5–23)	< 0.001
PODs 1–3 (early) drain removal	226 (29%)	217 (40%)	9 (4%)	< 0.001
PODs 4–5 drain removal	186 (24%)	142 (26%)	44 (18%)	0.006
Discharged with drains	94 (12%)	34 (6%)	60 (24%)	< 0.001
LOS (days), median (i.q.r.)	10 (7–17)	9 (7–13)	15 (9–25)	< 0.001
Readmission (30 days)	71 (9%)	38 (7%)	33 (13%)	0.006
Major morbidity	168 (21%)	82 (15%)	86 (34%)	< 0.001
90-day mortality	26 (3%)	13 (2%)	13 (5%)	0.043

Values are *n* (%) unless otherwise stated. POPF, postoperative pancreatic fistula; HR-PD, high-risk pancreatoduodenectomy; min, minutes; i.q.r., interquartile range; MPD, main pancreatic duct; ISGPS; International Study Group on Pancreatic Surgery; PPAP, post-pancreatectomy acute pancreatitis; POH; postoperative hyperamylasaemia; PPH, post-pancreatectomy haemorrhage; DGE, delayed gastric emptying; POD, postoperative day; LOS, length of hospital stay.

Whereas, during surgery, all HR-PD patients (251, 32%) were classified as ISGPS D (soft gland/MPD ≤ 3 mm), non-HR-PD patients (537, 68%) were classified as 327 (42%) ISGPS A (hard gland/MPD ≥ 3 mm), 103 (13%) ISGPS B (hard gland/MPD ≤ 3 mm), and 107 (13%) ISGPS C (soft gland/MPD ≥ 3 mm). During surgery, an ETS was positioned in 214 (85%) HR-PD patients, compared with 95 (18%) non-HR-PD patients (*P* < 0.001).

The overall POPF rate was 22% (173). The incidence of POPF was significantly higher in the HR-PD group (50% *versus* 9%; *P* < 0.001), as were the rates of PPAP (18% *versus* 2%; *P* < 0.001), PPH (18% *versus* 11%; *P* = 0.005), DGE (32% *versus* 18%; *P* < 0.001), intra-abdominal collections (deep surgical site infection) (39% *versus* 11%; *P* < 0.001) and sepsis (16% *versus* 7%; *P* < 0.001), percutaneous drainage (15% *versus* 5%; *P* < 0.001), relaparotomy (15% *versus* 7%; *P* < 0.001), unplanned intensive care unit admission (21% *versus* 9%; *P* < 0.001), and 90-day mortality (5% *versus* 2%; *P* = 0.043). Overall, the HR-PD group showed a longer median LOS (15 (i.q.r. 9–25) *versus* 9 (i.q.r. 7–13) days; *P* < 0.001), with significantly higher rates of major morbidity (34% *versus* 15%; *P* < 0.001).

Median POD of drain removal was significantly delayed in the HR-PD group (10 (i.q.r. 5–23) *versus* 4 (i.q.r. 3–6); *P* < 0.001), and 24% of HR-PD patients were discharged with drains, compared with 6% of non-HR-PD (*P* < 0.001). Early drain removal (PODs 1–3) was performed in 40% of non-HR-PD patients, compared with 4% of HR-PD patients (*P* < 0.001).

### Postoperative POPF predictors

The median values of each postoperative predictor across different PODs, stratified by POPF risk, are shown in *Table S2*, and a detailed report of missing values is shown in *Table S3*. The only variable with missing rates > 10% was POD 1 SL (94 (12%) missing).

Median DFA and SA/SL values were consistently higher in the HR-PD group across all PODs (all *P* < 0.001). However, in both the HR-PD and non-HR-PD groups, median DFA and SA/SL values decreased over time. Median C-RP was also higher in the HR-PD group (on PODs 3 and 5), though it followed a similar time-dependent trend, peaking on POD 3 in both groups.

**Fig. 2:**
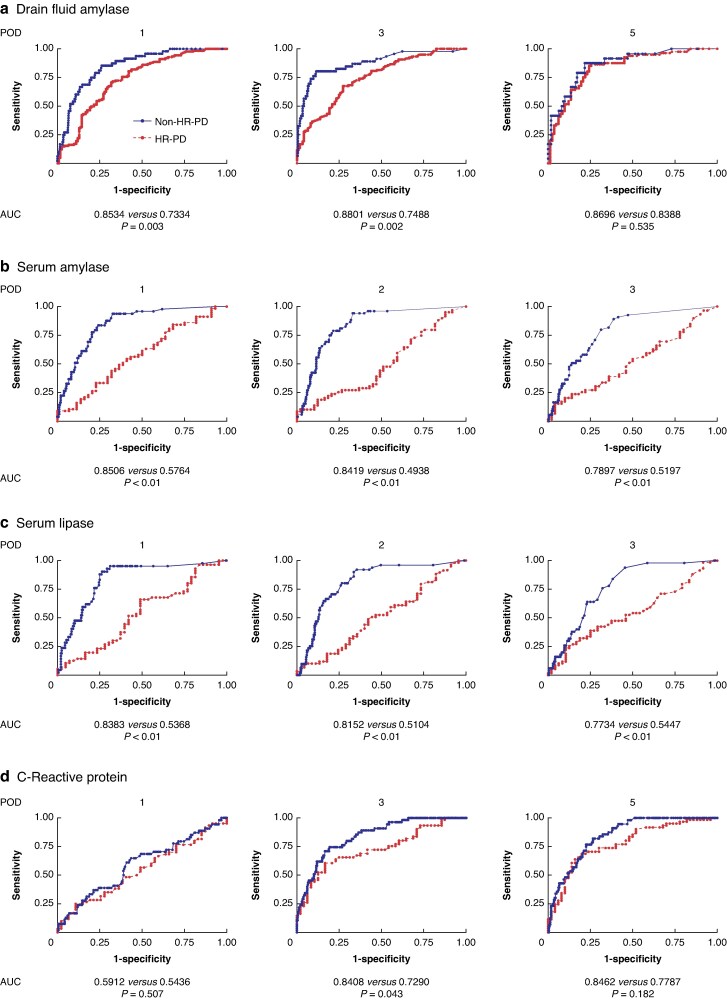
ROC curves and AUC of each predictor for POPF, stratified by POD and POPF risk **a** Drain fluid amylase, **b** serum amylase, **c** serum lipase, **d** C-reactive protein. ROC, receiver operating characteristic; AUC, area under the curve; POPF, postoperative pancreatic fistula; POD, postoperative day; HR-PD, high-risk pancreatoduodenectomy.

The diagnostic performance (ROC curves with AUC) of each clinical predictor—at different PODs and according to POPF risk—is displayed in *Fig. 2*. In the HR-PD group, DFA had a significantly lower AUC on POD 1 (AUC 0.73 *versus* 0.85; *P* = 0.003) and POD 3 (0.74 *versus* 0.88; *P* = 0.002) compared with the non-HR-PD group, but a comparable AUC on POD 5 (0.83 *versus* 0.86; *P* = 0.535). In the non-HR-PD group, SA/SL consistently had a higher AUC than in the HR-PD group (all *P* < 0.001). C-RP demonstrated similar ROC patterns in both the non-HR-PD and HR-PD groups, with the highest AUC on POD 5 (0.77 *versus* 0.84; *P* = 0.182).

The diagnostic performance of different predictor cut-offs for POPF across different PODs and according to POPF risk is shown in *Table S4*. In the non-HR-PD group, a DFA = 200 UI/l was identified as the best cut-off on POD 1 (SENS 85%, SPEC 74%, PPV 25%, NPV 98%, Prev+ 31%, Youden 0.593), whereas a DFA = 150 UI/l was identified as the best POD 3 cut-off (SENS 80%, SPEC 88%, PPV 40%, NPV 98%, Prev+ 18%, Youden 0.687). No DFA cut-offs were identified on POD 1 and 3 for the HR-PD group, whereas a DFA = 150 UI/ml was identified as the POD 5 best cut-off (SENS 86%, SPEC 72%, PPV 75%, NPV 84%, Prev+ 57%, Youden 0.584). The presence of SA and/or SL above the upper limit of normal on POD 1 was identified as the best cut-off for SA/SL in the non-HR-PD group (SENS 75%, SPEC 81%, PPV 49%, NPV 93%, Prev+ 30%, Youden 0.560). No cut-offs for SA/SL were identified in the HR-PD group. A C-RP = 150 mg/l was identified as the optimal cut-off on POD 5 in the HR-PD group (SENS 67%, SPEC 80%, PPV 80%, NPV 67%, Prev+ 46%, Youden 0.472).

A secondary analysis was performed to compare DFA median values and AUC between patients with and without an ETS (*Table S5* and *Fig. S1*), with similar results between HR-PD patients and patients with an ETS.

### Validation of DFA predictive ability and cut-offs

The predictive ability of DFA for HR *versus* non-HR-PD and the previously defined best DFA cut-offs were validated in a retrospective cohort of 398 patients, of whom 113 (28%) were classified as HR-PD (*Table S6*). In the validation cohort, POPF rates were 20% (56) in the non-HR-PD group *versus* 55% (62) in the HR-PD group. In the HR-PD group, DFA confirmed a significantly lower AUC on POD 1 (AUC 0.62 *versus* 0.86; *P* < 0.001) and POD 3 (0.65 *versus* 0.85; *P* < 0.001) compared with the non-HR-PD group, but a comparable AUC on POD 5 (0.74 *versus* 0.79; *P* = 0.384). In the non-HR-PD group, DFA = 200 UI/l was confirmed the best cut-off on POD 1 with a Youden index of 0.586 (SENS 87%, SPEC 72%, PPV 42%, NPV 96%), whereas DFA = 150 UI/l was the best cut-off on POD 3 with a Youden index of 0.558 (SENS 75%, SPEC 81%, PPV 48%, NPV 93%). In the HR-PD group, no DFA cut-off was identified on POD 1 and 3, whereas DFA = 150 UI/l was the best cut-off on POD 5 with a Youden index of 0.487 (SENS 66%, SPEC 83%, PPV 83%, NPV 66%).

### Dynamic drain management algorithm: criteria for early *versus* late drain removal

Criteria for early (POD 3) and late (POD 5) drain removal were selected, as shown in *Table 2*. Criteria for early drain removal in non-HR-PD patients were DFA < 200 UI/l on POD 1, DFA <150 UI/l on POD 3, and non-elevated SA and/or SL on POD 1. These combined criteria accurately predicted the absence of POPF in the non-HR-PD group (SENS 72%, SPEC 96%, PPV 99%, NPV 25%, Prev+ 66%, POPF 0.5%, Youden 0.674). Criteria for late drain removal in HR-PD patients were DFA < 150 UI/l and C-RP < 150 mg/ml on POD 5. These combined criteria accurately predicted the absence of POPF in the HR-PD group (SENS 64%, SPEC 95%, PPV 91%, NPV 77%, Prev+ 31%, POPF 9%, Youden 0.593).

**Table 2:** Diagnostic performances for POPF absence, for POD 3 and POD 5 drain removal criteria, according to POPF risk

	Criteria	SENS	SPEC	PPV	NPV	Prev+	POPF	Youden
**Non-HR-PD**								
POD 3 drain removal	POD 1 DFA < 200 UI/l; POD 1 SA/SL not elevated; POD 3 DFA < 150 U/l	72%	96%	99%	25%	66%	0.5%	0.674
**HR-PD**								
POD 5 drain removal	POD 5 DFA < 150 UI/l; POD 5 C-RP < 150 mg/l	64%	95%	91%	77%	31%	9%	0.593

POPF, postoperative pancreatic fistula; POD; postoperative day; SENS, sensitivity; SPEC, specificity; PPV, positive predictive value; NPV, negative predictive value; Prev+, prevalence of positive cut-offs in the examined population; Youden, Youden’s index; HR-PD, high-risk pancreatoduodenectomy; DFA, drain fluid amylase; SA, serum amylase; SL, serum lipase; C-RP, c-reactive protein.

**Fig. 3:**
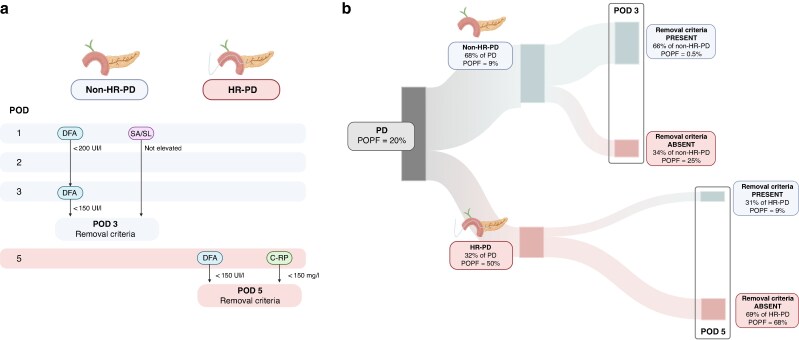
Proposal of tailored drain management protocol according to POPF risk, and Sankey diagram of drain removal criteria applied to HR-PD and non-HR-PD and POPF outcomes **a** Tailored drain management protocol according to POPF risk, **b** drain removal criteria applied to HR-PD, non-RH-PD, and POPF outcomes. POPF, postoperative pancreatic fistula; HR-PD, high-risk pancreatoduodenectomy; DFA, drain fluid amylase, SA/SL, serum amylase/lipase; POD, postoperative day; PD, pancreatoduodenectomy.

Based on these findings, a proposal for a tailored drain management protocol was developed, as shown in *Fig. 3a*. A Sankey diagram of drain removal criteria applied to HR-PD and non-HR-PD, with POPF outcomes, is displayed in *Fig. 3b*.

## Discussion

The present study proposed a novel, risk-based protocol for the postoperative drain management of patients undergoing PD with a specific focus on those at high risk for POPF. To do so, the diagnostic performance of different postoperative POPF predictors were evaluated dynamically, stratifying by POPF risk. In HR-PD patients, of whom 50% eventually developed POPF, DFA was not a reliable predictor in the early postoperative period (PODs 1–3), and neither SA/SL nor C-RP showed sufficient discriminatory ability. Therefore, whereas an early drain removal policy could be recommended in non-HR-PD patients based on DFA and SA/SL, HR-PD patients would require a delayed approach, with drain removal postponed until POD 5, when DFA and C-RP reach acceptable diagnostic accuracy.

The existence of a subgroup of patients with an intrinsically higher risk for POPF after PD is universally accepted. The intraoperative classification of such risk was first provided by the fistula risk score (FRS)^22^. Since then, it became clearer that POPF risk is mainly driven by pancreas-specific characteristics, such as gland texture and duct diameter^17^. Those parameters, on which the four-tier ISGPS classification is based, eventually represent surrogates of the secretive ability of the gland, and the technical difficulty of the anastomosis. Patients in the highest risk class (soft gland, main duct ≤ 3 mm), typically one-third of the total, have POPF rates between 30% and 40%^30–32^, increased risk of other concomitant pancreas-specific complications, and consequently worsened postoperative outcomes. Similarly, patients considered at higher risk during surgery in the present series experienced a five-fold increase in POPF rates compared with lower-risk patients. As expected, these patients also experienced higher rates of other pancreas-specific complications (including 18% of PPAP), major morbidity, and even mortality. However, the application of mitigation strategies (such as ETS) is a well recognized key element in reducing failure-to-rescue in this subgroup, as confirmed by the relatively low 90-day mortality, despite the dramatically high complications rates. The significant differences between HR-PD and non-HR-PD outcomes confirm the ability of currently available risk stratification tools to identify properly high-risk patients who can benefit from mitigation strategies.

Despite the vital importance of a tailored approach to these patients, a dynamic, multiparametric, risk-based assessment of postoperative drain management after HR-PD was missing. Some previous publications already explored tailored protocols based on intraoperative risk stratification, primarily relying on different DFA cut-offs adjusted for a patient’s risk level, generally adopting higher thresholds for higher-risk glands^8,9,11,12,33^. However, previous evaluations were rarely extended beyond POD 3, were limited to DFA without considering other predictors, and were not based on the most recent ISGPS risk classification. Two studies (Zureikat *et al*.^9^ and AlMasri *et al*.^33^) evaluated DFA kinetics between POD 1 and POD 3, reporting good predictive performance of a decreasing trajectory for excluding POPF. However, trajectories were not clearly stratified according to baseline POPF risk, and the overall model performance may have been driven by the higher prevalence of non-HR-PD (in which PODs 1–3 DFA have good predictive values) compared with HR-PD (where PODs 1–3 DFA have reduced predictive ability). Notably, a significant correlation between increasing FRS and higher POD 1/POD 3 DFA values was reported^9^, supporting the present findings.

Trans-anastomotic stents (internal or external) are considered optimal mitigation strategies for patients in the high-risk categories, to reduce not only POPF incidence but also the general and POPF-related morbidity burden, especially in the high-risk setting^23,34–40^. However, such mitigation strategies may also influence the accuracy and interpretation of traditional postoperative POPF predictors. Intuitively, the presence of a stent may significantly impact the predictive ability of DFA, potentially reducing its diagnostic performance. When an ETS is placed—diverting nearly all pancreatic juice outside the abdominal cavity—DFA activity may not reliably exclude the occurrence of POPF^13^. Interestingly, as showed by a dedicated subanalysis in the present study, median DFA activity was nonetheless higher in HR-PD patients and patients with a ETS, with poor prognostic performance, irrespective of subsequent development of POPF. Despite the external diversion of pancreatic juice, what seems to be the main driver of higher DFA values is the presence of a high-risk gland. In HR-PD, the DFA diagnostic performance increases only at POD 5, when DFA activity starts to decline in patients without POPF. Unfortunately, no other examined predictors were able to exclude POPF early enough in HR-PD patients to safely apply early drain removal policies. Even PODs 1–3 SA/SL activity were not useful early POPF predictors in HR-PD patients, as the prevalence of elevated SA/SL was very high in this group independently of POPF development. Finally, for both HR and non-HR patients, C-RP was confirmed to be a valuable indicator of POPF clinical relevance, but mostly from POD 3 onward, as a compendium of other more specific predictors^14^.

These results reinforce the importance of avoiding ‘blind’ early drain removal policies in high-risk patients, deferring drain removal to POD 5 according to DFA and C-RP levels. Conversely, in non-HR patients, PODs 1–3 DFA activity remains highly predictive and can safely drive early drain removal in most patients. When implemented with POD 1 SA/SL^28^, the predictive accuracy of the model might be further improved.

This study has some limitations. Most importantly, it represents an observational series in which postoperative decisions for HR-PD patients were not based on a predefined dedicated protocol, reflecting the absence of previous evidence and validated removal criteria in this group. Notably, despite an established institutional practice favouring DFA-based early drain removal, such policy was applied to only nine patients in the HR-PD group based on a very low drain volume output, reflecting a cautious surgical management, whereas it was maintained in almost half of the non-HR-PD patients. However, not performing early removal in the HR-PD group allowed comprehensive collection of dynamic postoperative data across PODs 1 to 5. A randomized clinical trial comparing early *versus* late removal policies in HR-PD patients would provide higher-level evidence, but the present analysis suggests that early drain removal is not supported by reliable indicators in this high-risk population, and might even be harmful given the high rate of POPF. On the other hand, in non-HR-PD patients with a low POPF risk and negative POD 1 DFA, a ‘super-early’ drain removal on POD 1 might be non-inferior—or even superior—relative to the conventional POD 3 removal. Despite this new policy not being represented in the present series, the present institutions are running a bicentric randomized clinical trial (FIDEL Trial: NCT06468917), which will hopefully bring new insights into early removal policies. When applied to the few selected patients with a negligible or low risk of fistula, even ‘drainless’ policies are described as safe by the literature^24^. However, drain omission was not part of the usual clinical practice in the present series. Finally, trans-anastomotic stents also include internal stents, widely used during minimally invasive surgery^41^ but excluded in the present analysis due to the relatively small sample size. It is not clear whether the results of the present analysis should also be applied to internal stents, given the macroscopic differences in their design and functioning. Future developments of the existing policies might come after the integration of novel tools into risk stratification, such as pathological data and artificial intelligence. For example, the acinar content calculated during the histologic assessment of pancreatic section margins was recently used to dichotomize retrospectively the risk of pancreas-specific complications into either high or low risk, surpassing the accuracy of existing intraoperative scores^32^. The diffusion of real-time, available pathological data during surgery may change traditional risk stratification.

In conclusion, postoperative drain management after PD remains challenging and warrants continuous refinement. Emerging approaches, such as the application of data-driven algorithms^16^, based on daily assessment of clinical and serological parameters and a proactive approach towards complications, have shown promising results in preventing the most severe sequelae of POPF, including mortality. However, tailored postoperative care should consider both the pancreas-specific risk of complications, and the use of available mitigations strategies like ETSs. In patients undergoing PD with a high intraoperative risk for POPF, early DFA and SA/SL activity are not reliable POPF predictors, and avoiding early drain removal—within the first 72 hours after surgery—appears more appropriate. In non-HR patients, early drain removal remains safe when guided by dynamic DFA and SA/SL assessment.

## Supplementary Material

zrag068_Supplementary_Data

## Data Availability

The data are not publicly available due to privacy restrictions, but are available from the corresponding author upon reasonable request
