# Supplementary material for: Drain management after pancreatoduodenectomy: risk-stratified dynamic algorithm
Source: BJS Open. 2026 Jun 26;10(3):zrag068. doi: 10.1093/bjsopen/zrag068 (PMC13308533; doi:10.1093/bjsopen/zrag068)
Supplement: zrag068_Supplementary_Data [file zrag068_supplementary_data.docx]

# **Drain Management after Pancreatoduodenectomy:**

# **A Risk-stratified Dynamic Algorithm**

Giampaolo Perri^1^, Elisa Bannone^2^, Isabella Frigerio^2,3^, Riccardo Pellegrini^1^, Martina Guerra^2^, Roberta Vella^2^, Alice Cattelani^2^, Elvira Adinolfi^2^, Umberto Cillo^1^,

Giovanni Butturini^2^* and Giovanni Marchegiani^1^*

^1^Hepato-pancreato-biliary and Liver Transplant Surgery Unit, Department of Surgical, Oncological and Gastroenterological Sciences (DiSCOG), University of Padua (Padua, Italy)

^2^Hepato-pancreato-biliary Surgery Department, Pederzoli Hospital (Peschiera Del Garda, Italy)

^3^Collegium Medicum, SAN University (Lodz, Poland)

* GB and GM share the last authorship

*Address correspondence and reprint requests to:*

Giovanni Marchegiani, MD, PhD

Chirurgia Epato-Bilio-Pancreatica e dei Trapianti di Fegato

Dipartimento di Scienze Chirurgiche Oncologiche e Gastroenterologiche - DISCOG

Università di Padova

Via Giustiniani 2, 35128 Padova

Email: [giovanni.marchegiani@unipd.it](mailto:giovanni.marchegiani@unipd.it)

ORCID ID: 0000-0002-6824-4533

X: Gio_Marchegiani

**Supplementary Materials - Index**

| **1) Supplementary Tables** | *pag. 2* |
| --- | --- |
| Supplementary Table 1 | *pag. 2* |
| Supplementary Table 2 | *pag. 4* |
| Supplementary Table 3 | *pag. 4* |
| Supplementary Table 4 | *pag. 5* |
| Supplementary Table 5 | *pag. 6* |
| Supplementary Table 6 | *pag. 7* |
| Supplementary Figure 1 | *pag. 8* |
| **2) Strobe Checklist** | *pag. 8* |
| **3) Supplementary Methods** | *pag. 10* |

**1) Supplementary Tables**

| **Supplementary Table 1. Preoperative, intraoperative and postoperative profile of included patients (N= 788), according to POPF risk** | | | | |
| --- | --- | --- | --- | --- |
| **Characteristics** | **Total, No. (%) (N= 788)** | **HR-PD, No. (%)** | | ***P*** |
|  |  | **No (N= 537, 68%)** | **Yes (N= 251, 32%)** |  |
| **Preoperative** | | | | |
| Institution, No. (%) |  |  |  | 0.112 |
| Pederzoli Hospital | 626 (79) | 435 (69) | 191 (31) |  |
| Padova University Hospital | 162 (21) | 102 (63) | 60 (37) |  |
| Age, median (iqr), y | 67 (59-74) | 68 (59-75) | 66 (59-73) | 0.060 |
| Female sex, No. (%) | 356 (45) | 242 (45) | 114 (45) | 0.926 |
| BMI, median (iqr) | 24 (22-27) | 24 (22-27) | 25 (22-27) | **0.018** |
| Active smoker, No. (%) | 158 (21) | 115 (22) | 43 (18) | 0.194 |
| Alchohol abuse, No. (%) | 38 (5) | 29 (6) | 9 (4) | 0.286 |
| Diabetes, No. (%) | 209 (27) | 168 (32) | 41 (17) | **<0.001** |
| Jaundice, No. (%) | 461 (59) | 332 (62) | 129 (52) | **0.006** |
| Janundice palliation, No. (%) |  |  |  | **0.001** |
| No | 329 (44) | 201 (40) | 128 (54) |  |
| Endoscopic stent | 393 (52) | 290 (56) | 103 (43) |  |
| Percutaneous drain | 31 (4) | 23 (4) | 8 (3) |  |
| Cardiovascoular comorbidities, No. (%) | 96 (13) | 69 (13) | 27 (11) | 0.402 |
| Hypertension, No. (%) | 389 (51) | 270 (52) | 119 (49) | 0.461 |
| Pulmonary comorbidities, No. (%) | 37 (5) | 19 (4) | 18 (7) | **0.024** |
| CKD, No. (%) | 31 (4) | 23 (4) | 8 (3) | 0.467 |
| Previous laparotomy, No. (%) | 281 (36) | 198 (37) | 83 (34) | 0.302 |
| ASA score ≥3, No. (%) | 216 (31) | 158 (33) | 58 (27) | 0.131 |
| Neoadjuvant therapy, No. (%) | 217 (28) | 185 (35) | 32 (13) | **<0.001** |
| Presumed diagnosis |  |  |  | **<0.001** |
| PDAC/chronic pancreatitis | 482 (61) | 393 (73) | 89 (35) |  |
| Duodenal/ampullary/cystic/NET | 306 (39) | 144 (27) | 162 (65) |  |
| **Intraoperative** |  |  |  |  |
| PD type, No. (%) |  |  |  | 0.106 |
| Pylorus-preserving | 677 (86) | 454 (85) | 223 (89) |  |
| Whipple | 111 (14) | 83 (15) | 28 (11) |  |
| Surgical approach, No. (%) |  |  |  | 0.057 |
| Open | 694 (88) | 481 (90) | 213 (85) |  |
| Robotic | 94 (12) | 56 (10) | 38 (15) |  |
| Pancreatic anastomosis, No. (%) |  |  |  | 0.462 |
| PJ | 776 (98) | 530 (99) | 246 (98) |  |
| PG | 12 (2) | 7 (1) | 5 (2) |  |
| ETS, No. (%) |  |  |  | **<0.001** |
| No | 479 (61) | 442 (82) | 37 (15) |  |
| Yes | 309 (39) | 95 (18) | 214 (85) |  |
| Vascular resection, No. (%) | 106 (13) | 84 (16) | 22 (9) | **0.008** |
| Operative time, median (iqr), min | 405 (335-475) | 400 (330-470) | 420 (340-480) | **0.048** |
| Blood loss, median (iqr), ml | 300 (200-400) | 300 (200-400) | 300 (200-450) | 0.882 |
| MPD size, median (iqr), mm | 4 (3-5) | 5 (4-6) | 2 (2-3) | **<0.001** |
| MPD size ≤ 3mm, No. (%) | 354 (45) | 103 (19) | 251 (100) | **<0.001** |
| Pancreatic texture, No. (%) |  |  |  | **<0.001** |
| Hard | 430 (55) | 430 (80) | _ |  |
| Soft | 358 (45) | 107 (20) | 251 (100) |  |
| ISGPS class, No. (%) |  |  |  | **<0.001** |
| A | 327 (42) | 327 (61) | _ |  |
| B | 103 (13) | 103 (19) | _ |  |
| C | 107 (13) | 107 (20) | _ |  |
| D | 251 (32) | _ | 251 (100) |  |
| **Postoperative** |  |  |  |  |
| POPF, No. (%) | 173 (22) | 48 (9) | 125 (50) | **<0.001** |
| POPF grade, No. (%) |  |  |  | **<0.001** |
| B | 143 (18) | 36 (7) | 107 (43) |  |
| C | 30 (4) | 12 (2) | 18 (7) |  |
| B/C PPAP, No. (%) | 57 (7) | 13 (2) | 44 (18) | **<0.001** |
| PPAP grade, No. (%) |  |  |  | **<0.001** |
| POH | 159 (20) | 59 (11) | 100 (40) |  |
| B | 39 (5) | 8 (1) | 31 (13) |  |
| C | 18 (2) | 5 (1) | 13 (5) |  |
| Intraabdominal collection (deep SSI), No. (%) | 155 (20) | 58 (11) | 97 (39) | **<0.001** |
| Biliary fistula, No. (%) | 37 (5) | 23 (4) | 14 (6) | 0.423 |
| DJ/GJ fistula, No. (%) | 20 (3) | 14 (3) | 6 (2) | 0.857 |
| Chylous fistula, No. (%) | 38 (5) | 32 (6) | 6 (2) | **0.029** |
| PPH, No. (%) | 105 (13) | 59 (11) | 46 (18) | **0.005** |
| PPH grade, No. (%) |  |  |  | **0.025** |
| A | 16 (2) | 11 (2) | 5 (2) |  |
| B | 46 (6) | 25 (5) | 21 (8) |  |
| C | 43 (5) | 23 (4) | 20 (8) |  |
| DGE, No. (%) | 180 (23) | 99 (18) | 81 (32) | **<0.001** |
| DGE grade, No. (%) |  |  |  | **<0.001** |
| A | 49 (6) | 34 (6) | 15 (6) |  |
| B | 73 (9) | 37 (7) | 36 (14) |  |
| C | 58 (7) | 28 (5) | 30 (12) |  |
| Sepsis, No. (%) | 75 (10) | 35 (7) | 40 (16) | **<0.001** |
| Percutaneous drainage, No. (%) | 63 (8) | 26 (5) | 37 (15) | **<0.001** |
| Transfusions, No. (%) | 183 (23) | 112 (21) | 71 (28) | **0.021** |
| Re-laparotomy, No. (%) | 75 (10) | 37 (7) | 38 (15) | **<0.001** |
| Postoperative pneumonia, No. (%) | 61 (8) | 33 (6) | 28 (11) | **0.014** |
| Respiratory insufficiency, No. (%) | 51 (6) | 21 (4) | 30 (12) | **<0.001** |
| Cardiovascoular complications, No. (%) | 45 (6) | 24 (4) | 21 (8) | **0.028** |
| Acute Kidney Injury, No. (%) | 19 (2) | 9 (2) | 10 (4) | **0.049** |
| Unplanned ICU admission, No. (%) | 99 (13) | 47 (9) | 52 (21) | **<0.001** |
| POD of drain removal, median (iqr), days | 5 (3-8) | 4 (3-6) | 10 (5-23) | **<0.001** |
| POD 1-3 (early) drain removal, No. (%) | 226 (29) | 217 (40) | 9 (4) | **<0.001** |
| POD 4-5 drain removal, No. (%) | 186 (24) | 142 (26) | 44 (18) | **0.006** |
| Discharged with drains, No. (%) | 94 (12) | 34 (6) | 60 (24) | **<0.001** |
| LOS, median (iqr), days | 10 (7-17) | 9 (7-13) | 15 (9-25) | **<0.001** |
| Readmission (30-days), No. (%) | 71 (9) | 38 (7) | 33 (13) | **0.006** |
| Clavien-Dindo, No (%) |  |  |  | **<0.001** |
| 0 | 221 (28) | 201 (38) | 20 (8) |  |
| 1 | 121 (15) | 88 (16) | 33 (13) |  |
| 2 | 278 (35) | 166 (31) | 112 (45) |  |
| 3 | 88 (11) | 46 (9) | 42 (17) |  |
| 4 | 54 (7) | 23 (4) | 31 (12) |  |
| 5 | 26 (3) | 13 (2) | 13 (5) |  |
| Major morbidity, No (%) | 168 (21) | 82 (15) | 86 (34) | **<0.001** |
| 90-days Mortality, No. (%) | 26 (3) | 13 (2) | 13 (5) | **0.043** |
| *Abbreviations: ISGPS, International Study Group on Pancreatic Surgery; BMI, body mass index; CKD, chronic kidney disease; ASA, American Society of Anesthesiologists Classification; PDAC, pancreatic ductal adenocarcinoma; NET, neuroendocrine tumor; PD, pancreatoduodenectomy; PJ, pancreatico-jejunostomy; PG, pancreatico-gastrostomy; ETS, exernal trans-anastomotic stent; MPD, main pancreatic duct; POPF, clinically relevant postoperative pancreatic fistula; BL, biochemical leak; POH; postoperative hyperamylasemia; PPAP, postopancreatectomy acute pancreatitis; DJ, duodeno-jejunal anastomosis; GJ, gastro-jejunal anastomosis; PPH, post pancreatectomy hemorrage; DGE, delayed gastrig emptying; ICU, intensive care unit; POD, postoperative day; LOS, lenght of hospital stay.* | | | | |

| **Supplementary Table 2. Median values for examined postoperative POPF predictor, according to POPF risk** | | | | |
| --- | --- | --- | --- | --- |
| **Predictors** | **Total, median (iqr) (N= 788)** | **HR-PD, median (iqr)** | | ***P*** |
|  |  | **No (N= 537)** | **Yes (N= 251)** |  |
| **DFA (UI/L)** | | | | |
| POD 1 | 216 (20-1915) | 48 (9-365) | 2487 (967-5756) | **<0.001** |
| POD 3 | 43 (4-410) | 8 (3-70) | 478 (157-1349) | **<0.001** |
| POD 5 | 95 (9-441) | 19 (3-224) | 215 (53-541) | **<0.001** |
| **SA (UI/L)** | | | | |
| POD 1 | 28 (4-97) | 10 (3-52) | 98 (47-165) | **<0.001** |
| POD 2 | 16 (3-67) | 8 (3-30) | 52 (21-186) | **<0.001** |
| POD 3 | 8 (3-29) | 5 (3-17) | 28 (15-56) | **<0.001** |
| **SL (UI/L)** | | | | |
| POD 1 | 35 (6-126) | 14 (5-58) | 116 (56-210) | **<0.001** |
| POD 2 | 17 (6-64) | 9 (5-34) | 60 (23-165) | **<0.001** |
| POD 3 | 11 (5-27) | 7 (5-17) | 31 (16-74) | **<0.001** |
| **C-RP** | | | | |
| POD 1 | 89 (65-114) | 89 (65-114) | 88 (67-116) | 0.893 |
| POD 3 | 187 (124-249) | 174 (115-234) | 218 (154-271) | **<0.001** |
| POD 5 | 104 (57-169) | 91 (44-157) | 141 (83-206) | **<0.001** |
| *Abbreviations: ISGPS, International Study Group on Pancreatic Surgery; POD, postoperative day; DFA, drain fluid amylase; SA, serum amylase; SL, serum lipase; C-RP, c-reactive protein.* | | | | |

| **Supplementary Table 3. Missing values for examined postoperative POPF indicators, according to POPF risk** | | | |
| --- | --- | --- | --- |
| **Missing values** | **Total No. (%) (N= 788)** | **HR-PD, No. (%)** | |
|  |  | **No (N= 537)** | **Yes (N= 251)** |
| **DFA (U/L)** | | | |
| POD 1 | 8 (1) | 6 (1) | 2 (1) |
| POD 3 | 26 (3) | 22 (4) | 6 (2) |
| POD 5 | 78 (10) | 43 (8) | 35 (14) |
| **SA (UI/L)** | | | |
| POD 1 | 68 (8) | 43 (8) | 25 (10) |
| POD 2 | 37 (5) | 27 (5) | 10 (4) |
| POD 3 | 15 (2) | 5 (1) | 10 (4) |
| **SL (UI/L)** | | | |
| POD 1 | 94 (12) | 64 (12) | 30 (12) |
| POD 2 | 47 (6) | 27 (5) | 20 (8) |
| POD 3 | 40 (5) | 27 (5) | 13 (5) |
| **C-RP** | | | |
| POD 1 | 28 (3) | 16 (3) | 12 (5) |
| POD 3 | 8 (1) | 5 (1) | 3 (8) |
| POD 5 | 10 (1) | 5 (1) | 5 (2) |
| *Abbreviations: ISGPS, International Study Group on Pancreatic Surgery; POD, postoperative day; DFA, drain fluid amylase; SA, serum amylase; SL, serum lipase; C-RP, c-reactive protein.* | | | |

| **Supplementary Table 4**. Diagnostic performances (for POPF) of different predictors cutoffs at different PODs, according to POPF risk (best performing cutoffs are highlighted). | | | | | | | | | | | | | |
| --- | --- | --- | --- | --- | --- | --- | --- | --- | --- | --- | --- | --- | --- |
|  |  | **HR-PD** | | | | | | | | | | | |
| **POD** | **PREDICTORS** | **No** | | | | | | **Yes** | | | | | |
| **1** | **DFA (IU/L)** | **SENS** | **SPEC** | **PPV** | **NPV** | **Prev +** | **Youden** | **SENS** | **SPEC** | **PPV** | **NPV** | **Prev +** | **Youden** |
|  | 5000 | 25% | 97% | 43% | 93% | 5% | 0.217 | 42% | 86% | 74% | 60% | 28% | 0.280 |
|  | 3000 | 27% | 95% | 34% | 93% | 7% | 0.219 | 63% | 71% | 68% | 67% | 46% | 0.348 |
|  | 2500 | 31% | 94% | 34% | 93% | 8% | 0.252 | 67% | 68% | 67% | 68% | 49% | 0.357 |
|  | 2000 | 44% | 94% | 40% | 94% | 10% | 0.373 | 76% | 62% | 66% | 72% | 57% | 0.375 |
|  | 1500 | 52% | 91% | 37% | 95% | 13% | 0.434 | 80% | 56% | 64% | 75% | 62% | 0.368 |
|  | 1000 | 60% | 89% | 35% | 95% | 15% | 0.494 | 89% | 40% | 59% | 78% | 74% | 0.291 |
|  | 500 | 69% | 83% | 29% | 96% | 21% | 0.520 | 98% | 25% | 56% | 91% | 86% | 0.222 |
|  | 300 | 77% | 78% | 26% | 97% | 27% | 0.551 | 98% | 17% | 54% | 91% | 91% | 0.150 |
|  | **200** | 85% | 74% | 25% | 98% | 31% | 0.593 | 99% | 13% | 53% | 94% | 93% | 0.127 |
|  | 150 | 85% | 69% | 22% | 98% | 36% | 0.546 | 100% | 12% | 53% | 100% | 94% | 0.119 |
|  | **SA/SL (IU/L)** | **SENS** | **SPEC** | **PPV** | **NPV** | **Prev +** | **Youden** | **SENS** | **SPEC** | **PPV** | **NPV** | **Prev +** | **Youden** |
|  | Elevated SA (>53 UI/L) | 61% | 83% | 46% | 90% | 24% | 0.447 | 74% | 36% | 60% | 52% | 69% | 0.100 |
|  | Elevated SL (>60 UI/L) | 62% | 83% | 43% | 92% | 24% | 0.452 | 73% | 26% | 56% | 42% | 74% | 0.000 |
|  | **Elevated SA and/or SL** | 75% | 81% | 49% | 93% | 30% | 0.560 | 76% | 26% | 56% | 46% | 75% | 0.018 |
|  | **C-RP (mg/L)** | **SENS** | **SPEC** | **PPV** | **NPV** | **Prev +** | **Youden** | **SENS** | **SPEC** | **PPV** | **NPV** | **Prev +** | **Youden** |
|  | 80 | 70% | 43% | 23% | 86% | 59% | 0.134 | 63% | 43% | 58% | 48% | 61% | 0.059 |
|  | 110 | 39% | 74% | 27% | 83% | 28% | 0.131 | 32% | 74% | 61% | 46% | 29% | 0.061 |
|  | 130 | 24% | 86% | 29% | 82% | 16% | 0.096 | 23% | 89% | 74% | 48% | 18% | 0.127 |
| **2** | **SA/SL (IU/L)** | **SENS** | **SPEC** | **PPV** | **NPV** | **Prev +** | **Youden** | **SENS** | **SPEC** | **PPV** | **NPV** | **Prev +** | **Youden** |
|  | Elevated SA (>53 UI/L) | 56% | 89% | 54% | 89% | 19% | 0.444 | 47% | 49% | 53% | 44% | 49% | 0.000 |
|  | Elevated SL (>60 UI/L) | 43% | 90% | 49% | 87% | 16% | 0.327 | 51% | 53% | 59% | 45% | 49% | 0.042 |
|  | Elevated SA and/or SL | 57% | 88% | 53% | 90% | 21% | 0.454 | 57% | 47% | 57% | 47% | 55% | 0.036 |
| **3** | **DFA (IU/L)** | **SENS** | **SPEC** | **PPV** | **NPV** | **Prev +** | **Youden** | **SENS** | **SPEC** | **PPV** | **NPV** | **Prev +** | **Youden** |
|  | 600 | 57% | 95% | 52% | 96% | 10% | 0.514 | 60% | 75% | 70% | 66% | 42% | 0.352 |
|  | 500 | 59% | 94% | 49% | 96% | 11% | 0.527 | 68% | 70% | 68% | 69% | 49% | 0.371 |
|  | 400 | 65% | 94% | 50% | 96% | 12% | 0.588 | 73% | 63% | 65% | 71% | 54% | 0.357 |
|  | 300 | 70% | 92% | 47% | 97% | 13% | 0.619 | 81% | 53% | 62% | 74% | 64% | 0.336 |
|  | 250 | 74% | 92% | 47% | 97% | 14% | 0.655 | 83% | 49% | 61% | 75% | 67% | 0.321 |
|  | 200 | 76% | 90% | 42% | 97% | 16% | 0.656 | 86% | 46% | 61% | 77% | 69% | 0.322 |
|  | **150** | 80% | 88% | 40% | 98% | 18% | 0.687 | 91% | 38% | 58% | 81% | 76% | 0.284 |
|  | **SA/SL (IU/L)** | **SENS** | **SPEC** | **PPV** | **NPV** | **Prev +** | **Youden** | **SENS** | **SPEC** | **PPV** | **NPV** | **Prev +** | **Youden** |
|  | Elevated SA (>53 UI/L) | 16% | 94% | 41% | 82% | 8% | 0.106 | 29% | 72% | 55% | 46% | 28% | 0.008 |
|  | Elevated SL (>60 UI/L) | 12% | 96% | 40% | 83% | 5% | 0.079 | 37% | 75% | 65% | 49% | 32% | 0.123 |
|  | Elevated SA and/or SL | 18% | 94% | 42% | 83% | 8% | 0.121 | 39% | 73% | 63% | 50% | 34% | 0.119 |
|  | **C-RP (mg/L)** | **SENS** | **SPEC** | **PPV** | **NPV** | **Prev +** | **Youden** | **SENS** | **SPEC** | **PPV** | **NPV** | **Prev +** | **Youden** |
|  | 200 | 76% | 72% | 40% | 93% | 37% | 0.487 | 72% | 50% | 65% | 59% | 62% | 0.221 |
|  | 230 | 62% | 84% | 50% | 91% | 26% | 0.510 | 66% | 75% | 77% | 63% | 48% | 0.406 |
|  | 250 | 58% | 89% | 55% | 90% | 20% | 0.468 | 52% | 85% | 82% | 58% | 35% | 0.379 |
| **5** | **DFA (IU/L)** | **SENS** | **SPEC** | **PPV** | **NPV** | **Prev +** | **Youden** | **SENS** | **SPEC** | **PPV** | **NPV** | **Prev +** | **Youden** |
|  | 600 | 54% | 91% | 57% | 90% | 17% | 0.452 | 41% | 93% | 85% | 61% | 24% | 0.334 |
|  | 500 | 54% | 90% | 54% | 90% | 18% | 0.443 | 44% | 91% | 84% | 63% | 26% | 0.359 |
|  | 400 | 58% | 88% | 52% | 91% | 20% | 0.466 | 52% | 89% | 82% | 65% | 31% | 0.409 |
|  | 300 | 63% | 86% | 50% | 91% | 22% | 0.490 | 64% | 84% | 80% | 70% | 40% | 0.483 |
|  | 250 | 67% | 86% | 52% | 92% | 23% | 0.532 | 68% | 80% | 77% | 72% | 44% | 0.484 |
|  | 200 | 75% | 83% | 49% | 94% | 27% | 0.579 | 80% | 76% | 76% | 79% | 52% | 0.559 |
|  | **150** | 79% | 79% | 45% | 95% | 31% | 0.584 | 86% | 72% | 75% | 84% | 57% | 0.584 |
|  | **C-RP (mg/L)** | **SENS** | **SPEC** | **PPV** | **NPV** | **Prev +** | **Youden** | **SENS** | **SPEC** | **PPV** | **NPV** | **Prev +** | **Youden** |
|  | 100 | 88% | 62% | 37% | 95% | 48% | 0.499 | 80% | 54% | 68% | 69% | 65% | 0.343 |
|  | 130 | 77% | 77% | 45% | 93% | 34% | 0.533 | 74% | 70% | 75% | 69% | 54% | 0.438 |
|  | **150** | 64% | 82% | 47% | 90% | 27% | 0.461 | 67% | 80% | 80% | 67% | 46% | 0.472 |
| *Abbreviations: ISGPS, International Study Group on Pancreatic Surgery; POD, postoperative day; DFA, drain fluid amylase; SA, serum amylase; SL, serum lipase; C-RP, c-reactive protein; SENS, sensitivity; SPEC, specificity; PPV, positive predictive value; NPV, negative predictive value; Prev +, prevalence of positive cut-offs in the examined population; Youden, Youden's index.* | | | | | | | | | | | | | |

| **Supplementary Table 5. Median values for DFA, in patients with and without ETS** | | | | |
| --- | --- | --- | --- | --- |
| **Indicators** | **Total, median (iqr)**  **(N= 788)** | **ETS, median (iqr)** | | ***P*** |
|  |  | **No (N= 479)** | **Yes (N= 309)** |  |
| **DFA (U/L)** | | | | |
| POD 1 | 216 (20-1915) | 43 (9-364) | 1818 (475-4854) | **<0.001** |
| POD 3 | 43 (4-410) | 7 (3-68) | 323 (76-957) | **<0.001** |
| POD 5 | 95 (9-441) | 55 (4-265) | 145 (19-521) | **<0.001** |
| *Abbreviations: ETS, externalized trans-anastomotic stent; POD, postoperative day; DFA, drain fluid amylase.* | | | | |

| **Supplementary Table 6. Preoperative, intraoperative and postoperative characteristics of the validation cohort (N= 398), according to POPF risk** | | | | |
| --- | --- | --- | --- | --- |
| **Characteristics** | **Total, No. (%)**  **(N= 398)** | **HR-PD, No. (%)** | | ***P*** |
|  |  | **No (N= 285, 72%)** | **Yes (N= 113, 28%)** |  |
| **Preoperative** | | | | |
| Institution, No. (%) |  |  |  | 0.494 |
| Pederzoli Hospital | 297 (75) | 210 (71) | 87 (29) |  |
| Padova University Hospital | 101 (25) | 75 (74) | 26 (26) |  |
| Age, median (iqr), y | 66 (57-72) | 66 (58-72) | 64 (53-71) | **0.013** |
| Female sex, No. (%) | 187 (47) | 145 (51) | 42 (37) | **0.013** |
| BMI, median (iqr) | 24 (22-27) | 24 (22-27) | 25 (22-27) | **0.056** |
| **Intraoperative** | | | | |
| PD type, No. (%) |  |  |  | 0.566 |
| Pylorus-preserving | 324 (81) | 230 (81) | 94 (83) |  |
| Whipple | 74 (19) | 55 (19) | 19 (17) |  |
| Vascular resection, No. (%) | 56 (14) | 46 (16) | 10 (9) | 0.059 |
| Blood loss, median (iqr), ml | 600 (600-900) | 600 (300-910) | 500 (250-800) | 0.077 |
| ISGPS class, No. (%) |  |  |  |  |
| A | 140 (35) | 140 (49) | _ |  |
| B | 61 (15) | 61 (21) | _ |  |
| C | 84 (21) | 84 (29) | _ |  |
| D | 113 (28) | _ | 113 (100) |  |
| ETS, No. (%) |  |  |  | **<0.001** |
| Yes | 163 (41) | 62 (22) | 101 (89) |  |
| No | 235 (59) | 223 (78) | 12 (11) |  |
| Pancreatic anastomosis, No. (%) |  |  |  | 0.135 |
| PJ | 381 (96) | 277 (97) | 104 (92) | **0.022** |
| PG | 17 (4) | 8 (3) | 9 (8) |  |
| **Postoperative** | | | | |
| POPF, No. (%) | 118 (30) | 56 (20) | 62 (55) | **<0.001** |
| POPF grade, No. (%) |  |  |  | 0.176 |
| B | 107 (27) | 52 (18) | 55 (48) |  |
| C | 11 (3) | 4 (1) | 7 (6) |  |
| PPH, No. (%) | 48 (12) | 28 (10) | 20 (18) | **0.030** |
| DGE, No. (%) | 64 (16) | 32 (11) | 32 (28) | **<0.001** |
| POD 1-3 (early) drain removal, No. (%) | 119 (30) | 115 (40) | 4 (4) | **<0.001** |
| Major morbidity, No (%) | 65 (16) | 35 (12) | 30 (27) | **<0.001** |
| Mortality, No. (%) | 8 (2) | 6 (2) | 2 (2) | 0.830 |
| *Abbreviations: BMI, body mass index; PD, pancreatoduodenectomy; ETS, exernal trans-anastomotic stent; PJ, pancreatico-jejunostomy; PG, pancreatico-gastrostomy; POPF, clinically relevant postoperative pancreatic fistula; BL, biochemical leak; POH; postoperative hyperamylasemia; PPAP, postpancreatectomy acute pancreatitis; PPH, post pancreatectomy hemorrage; DGE, delayed gastrig emptying; POD, postoperative day.* | | | | |

**Supplementary Figure 1.** ROC curves and AUC of DFA, stratified by POD and ETS presence.

**
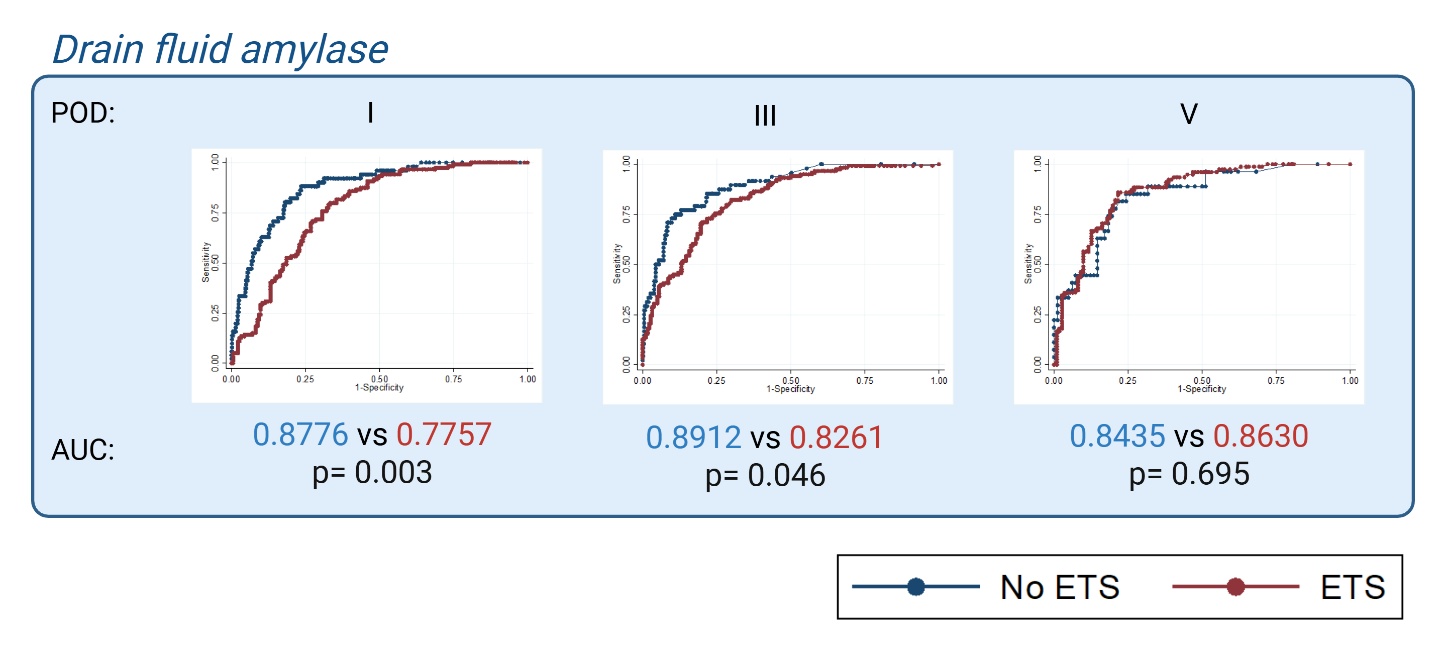
**

*(ROC, Receiver operating characteristic; AUC, area under the curve; POD, postoperative day; ETS, externalized trans-anastomotic stent)*

**2) STROBE checklist of items that should be included in reports of observational studies**

|  | **Item No** | **Recommendation** | **Page** |
| --- | --- | --- | --- |
| Title and abstract | 1 | (*a*) Indicate the study’s design with a commonly used term in the title or the abstract | 1 |
|  |  | (*b*) Provide in the abstract an informative and balanced summary of what was done and what was found | 2 |
| Introduction | | |  |
| Background/rationale | 2 | Explain the scientific background and rationale for the investigation being reported | 3 |
| Objectives | 3 | State specific objectives, including any prespecified hypotheses | 3 |
| Methods | | |  |
| Study design | 4 | Present key elements of study design early in the paper | 4 |
| Setting | 5 | Describe the setting, locations, and relevant dates, including periods of recruitment, exposure, follow-up, and data collection | 4 |
| Participants | 6 | (*a*) *Cohort study*—Give the eligibility criteria, and the sources and methods of selection of participants. Describe methods of follow-up | 4-5 |
|  |  | (*b*) *Cohort study*—For matched studies, give matching criteria and number of exposed and unexposed | 4-5 |
| Variables | 7 | Clearly define all outcomes, exposures, predictors, potential confounders, and effect modifiers. Give diagnostic criteria, if applicable | 4-5 |
| Data sources/ measurement | 8* | For each variable of interest, give sources of data and details of methods of assessment (measurement). Describe comparability of assessment methods if there is more than one group | *4-5* |
| Bias | 9 | Describe any efforts to address potential sources of bias | 4-5 |
| Study size | 10 | Explain how the study size was arrived at | 4-5 |
| Quantitative variables | 11 | Explain how quantitative variables were handled in the analyses. If applicable, describe which groupings were chosen and why | 4-5 |
| Statistical methods | 12 | (*a*) Describe all statistical methods, including those used to control for confounding | 5 |
|  |  | (*b*) Describe any methods used to examine subgroups and interactions | 5 |
|  |  | (*c*) Explain how missing data were addressed | 5 |
|  |  | (*d*) *Cohort study*—If applicable, explain how loss to follow-up was addressed  *Case-control study*—If applicable, explain how matching of cases and controls was addressed  *Cross-sectional study*—If applicable, describe analytical methods taking account of sampling strategy | 5 |
|  |  | (*e*) Describe any sensitivity analyses | 5 |

| Results | | |  |
| --- | --- | --- | --- |
| Participants | 13* | (a) Report numbers of individuals at each stage of study—eg numbers potentially eligible, examined for eligibility, confirmed eligible, included in the study, completing follow-up, and analysed | 6-9 |
|  |  | (b) Give reasons for non-participation at each stage | 6-9 |
|  |  | (c) Consider use of a flow diagram | Figure 1 |
| Descriptive data | 14* | (a) Give characteristics of study participants (eg demographic, clinical, social) and information on exposures and potential confounders | 6-9 |
|  |  | (b) Indicate number of participants with missing data for each variable of interest | 6-9 |
|  |  | (c) *Cohort study*—Summarise follow-up time (eg, average and total amount) | 6-9 |
| Outcome data | 15* | *Cohort study*—Report numbers of outcome events or summary measures over time | *6-9* |
|  |  | *Case-control study—*Report numbers in each exposure category, or summary measures of exposure | *6-9* |
|  |  | *Cross-sectional study—*Report numbers of outcome events or summary measures | *6-9* |
| Main results | 16 | (*a*) Give unadjusted estimates and, if applicable, confounder-adjusted estimates and their precision (eg, 95% confidence interval). Make clear which confounders were adjusted for and why they were included | 6-9 |
|  |  | (*b*) Report category boundaries when continuous variables were categorized | 6-9 |
|  |  | (*c*) If relevant, consider translating estimates of relative risk into absolute risk for a meaningful time period | 6-9 |
| Other analyses | 17 | Report other analyses done—eg analyses of subgroups and interactions, and sensitivity analyses | 6-9 |
| Discussion | | |  |
| Key results | 18 | Summarise key results with reference to study objectives | 9-12 |
| Limitations | 19 | Discuss limitations of the study, taking into account sources of potential bias or imprecision. Discuss both direction and magnitude of any potential bias | 9-12 |
| Interpretation | 20 | Give a cautious overall interpretation of results considering objectives, limitations, multiplicity of analyses, results from similar studies, and other relevant evidence | 9-12 |
| Generalisability | 21 | Discuss the generalisability (external validity) of the study results | 9-12 |
| Other information | | |  |
| Funding | 22 | Give the source of funding and the role of the funders for the present study and, if applicable, for the original study on which the present article is based | 13 |

**3) Supplementary Methods**

*Inclusion Criteria*

- Age ≥18
- Undergoing pancreaticoduodenectomy
- Available DFA measurement on POD 1 and/or 3

*Exclusion Critera*

- Intraoperative ISGPS POPF risk stratification not available
- Internal trans-anastomotic stent positioning
- DFA measurement not available in POD 1 and 3
